# Local Adaptation and Osmoregulatory Mechanisms of the Copepod *Acartia tonsa* Under Low Salinity Stress

**DOI:** 10.1111/mec.70208

**Published:** 2025-12-14

**Authors:** Alexandra Hahn, Jennifer C. Nascimento‐Schulze, Georgia Avgerinou, Till Bayer, Reid S. Brennan

**Affiliations:** ^1^ GEOMAR Helmholtz Centre for Ocean Research Kiel Kiel Germany

**Keywords:** *Acartia tonsa*, copepod, gene expression, local adaptation, osmoregulation, salinity tolerance

## Abstract

Salinity—an essential factor shaping marine species distributions—is rapidly shifting due to global change, yet the mechanisms of salinity tolerance and adaptation remain poorly understood. We investigated local adaptation in the calanoid copepod *Acartia tonsa* , a broadly distributed marine species that thrives in the brackish Baltic Sea. Using a common‐garden design, we compared physiological and transcriptomic responses to low salinity between populations from the North Sea (> 25 PSU) and the Baltic Sea (< 15 PSU). Baltic copepods exhibited significantly higher survival under low salinity, indicating local adaptation. While both populations shared a core osmoregulatory strategy involving active ion transport and regulation of amino acids, transcriptomic profiles revealed population‐specific differences. Baltic individuals showed a reduced overall gene expression response, yet maintained higher relative expression of osmoregulatory genes—suggesting a more efficient and primed transcriptomic response. In contrast, North Sea copepods exhibited broader transcriptional shifts, including downregulation of metabolic and developmental pathways after prolonged stress exposure, possibly reflecting energy conservation mechanisms. These findings reveal that *A. tonsa* possesses both a plastic osmoregulatory strategy and population‐level adaptation that enable survival in extreme salinity conditions. While both populations tolerate short‐term exposure to low salinity, local adaptation has enhanced the Baltic population's resilience. This suggests that *A. tonsa* is broadly tolerant of moderate climate‐driven salinity declines across most of its distribution. However, our data also indicate potential range contractions in the lowest salinity zones of the Baltic Sea, underscoring the importance of identifying physiological and genetic thresholds in climate resilience studies.

## 1 Introduction

Salinity plays an important role in shaping marine ecosystems as it poses a barrier for dispersal, restricting species distribution. Transitioning from marine conditions to brackish or even fresh water forces marine organisms to either actively transport ions across their cell membrane to maintain a higher internal osmolarity, making them osmoregulators, or to lower their osmolarity to be in equilibrium with the environment, making them osmoconformers (Mantel and Farmer 1983). If an organism is unable to react to decreases in salinity, the resulting influx of water can lead to physiological impairment and potentially death (Lange 1970). Given this, in habitats with strong salinity gradients such as estuaries, organisms face challenging conditions (Lawrence et al. 2004). When environmental clines, including salinity, are stable over longer time scales, selection pressure can lead populations to adapt to local salinity conditions, giving them a heritable fitness advantage in this specific habitat (Kawecki and Ebert 2004). Alternatively, when the environment fluctuates within an organism's lifetime, plasticity is expected to evolve (Bitter et al. 2021).

Understanding if and how organisms can tolerate environmental stressors such as low salinity, along with assessing their capacity for local adaptation, helps us predict species dispersal, evaluate their potential to invade new environments, and anticipate how organisms may respond to environmental shifts driven by climate change. Though climate change is unanimously regarded as a threat to biodiversity by the scientific community, not all effects are researched equally. While there is extensive literature on ocean warming, ocean acidification and ocean deoxygenation (Garcia‐Soto et al. 2021; Gruber et al. 2021) and the resulting implications for marine life (Deutsch et al. 2024; Leung et al. 2022; Venegas et al. 2023), the consequences of changes in salinity are widely under‐researched (Lee, Downey, et al. 2022). Even seemingly minor salinity reductions can push organisms to their tolerance limit and constrain species distribution if environmental salinity drops below the critical salinity threshold separating marine and freshwater organisms (Khlebovich and Abramova 2000). In both low and high latitudes, salinity is predicted to decline due to increased river runoff, melting of glaciers, increased precipitation and changes in ocean fluxes (Du et al. 2019; McCrystall et al. 2021; Skliris et al. 2014). Therefore, understanding the effects of salinity on organisms and populations is crucial for predicting how global change will shape marine habitats.

The copepod *Acartia tonsa* (Dana 1849), is an ideal species to understand local adaptation and responses to changes in salinity. This species is a predominantly coastal calanoid copepod with a global distribution, attributed to its euryhalinity and high thermal tolerance (Cervetto et al. 1999; Svetlichny and Hubareva 2014; Walter and Boxshall 2024). Copepods, including *A. tonsa* , are an integral component of marine food webs, serving as a prey item for commercially important fish species (Mauchline 1998), linking primary production to higher trophic levels (Turner 2004). Because *A. tonsa* inhabits coastal environments, it experiences rapid salinity fluctuations within its lifespan and may harbour broad plasticity to tolerate these changes. However, *A. tonsa* also exists along steep salinity gradients, such as the Baltic Sea, a unique ecosystem with a strong east–west salinity gradient from fully marine to nearly fresh water (Szymczycha et al. 2019). There is evidence that *A. tonsa* is not native to European waters and was introduced to the North Sea in the early 1900s before colonising the Baltic shortly after (Brylinski 1981; Ojaveer et al. 2016; Smirnov 1935). Since then, the species has swiftly established itself as a dominant zooplankton species in the warm summer months (Redeke 1934). Alternatively, the species may not have been correctly identified prior to the 1900s and could have been present in the Baltic Sea for longer periods. The Baltic Sea has only been connected to the North Sea for ~8000 years (Björck 1995), representing the earliest time that it could have been colonised by *A. tonsa* . In either scenario, given *A. tonsa* 's rapid generation time, populations likely experience differences in the mean salinity along this cline and may be locally adapted.

In addition to the strong salinity gradient in the Baltic Sea, its enclosed nature also makes it especially vulnerable to climate change impacts (Reusch et al. 2018). While there is some uncertainty in salinity forecasting (Meier et al. 2022), sea surface salinities in the Baltic Sea have decreased by up to 1.14 PSU over the last six decades (Kankaanpää et al. 2023) and are predicted to further decrease over the next century (Saraiva et al. 2019), posing a challenge for many Baltic species. For a small number of species in the Baltic Sea, including phytoplankton (Pinseel et al. 2025), mussels (Knöbel et al. 2021) and fish (Guo et al. 2016), adaptation to local salinity has been shown, suggesting that some species possess the capacity to adapt to salinity reductions in this region. However, the resilience and adaptive capacity to changing salinity for most species in the Baltic Sea, including copepods, is still unknown.

Previous transcriptomic analyses aiming to uncover the osmoregulatory strategy of copepods have primarily focused on two species, *Eurytemora affinis* —a calanoid copepod known for its capacity to invade freshwater habitats (Gerber et al. 2016)—and *Tigriopus californicus* —a harpacticoid copepod that inhabits tidal pools subjected to extreme temperature and salinity fluctuations (DeBiasse et al. 2018). However, both of these species are relatively restricted in their distributions: *E. affinis* to low salinity brackish regions and *T. californicus* to coastal tide pools. In contrast, *A. tonsa* occupies coastal regions generally with few barriers to dispersal, providing an ideal system to understand the largely unexplored mechanisms of osmoregulation and the presence of salinity local adaptation in a widely distributed pelagic copepod.

Here, we fill this gap using *A. tonsa* from the Baltic and North Sea to reveal mechanisms of local adaptation and the core mechanisms of osmoregulation at low salinities. We combined transcriptomics with phenotypic data to understand (1) the conserved osmoregulatory mechanisms of *A. tonsa* enabling tolerance of low salinity stress, (2) whether there is local adaptation to low salinity between populations originating from the brackish Baltic Sea compared to the fully marine North Sea and (3) the implications for the capacity of *A. tonsa* to tolerate or further adapt to future changes in ocean salinity. By combining transcriptomic data with fitness measurements, we provide a holistic picture of the effects of low salinity on *A. tonsa* from unique osmotic environments.

## 2 Methods

### 2.1 Sampling and Culturing

**FIGURE 1:**
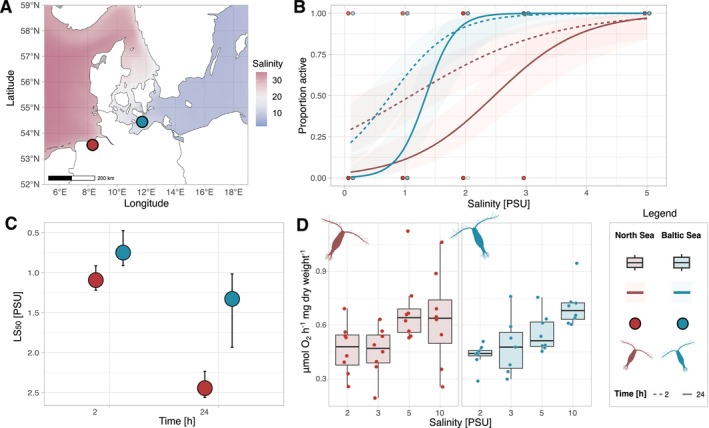
Fitness measurements for Baltic (labelled in blue) and North Sea (labelled in red) *Acartia tonsa* populations. (A) Map of sampling locations. Salinity data from CMEMS https://doi.org/10.48670/moi‐00021. (B) Acute survival of adult females at low salinities for 2 h (dashed line) and 24 h (solid line); points represent observations, and colour intensity represents number of observed individuals. (C) LS50 values for acute survival shown in B, error bars show distribution of replicates. (D) Oxygen consumption of acutely exposed animals (2‐h acclimation) in μmol O_2_ per hour normalised for dried body weight.

*Acartia tonsa* populations for this study were collected by 100 μm WP2 net hauls in the North Sea at Wilhelmshaven (WH: 53°30′46.8″ N 8°08′56.4″ E, collected on Sept 09th 2022) hereafter referred to as North Sea, and in the Western Baltic Sea (SW08: 54°24′46.8″ N 11°37′01.2″ E, collected on Aug 31st 2022) hereafter referred to as Baltic Sea (Figure 1A). Daily measurements from E.U. Copernicus Marine Service Information (CMEMS) from 1993 to 2021 showed that salinity conditions were significantly different between these stations (*p* < 0.001) with the mean surface salinity during the summer months being 26.2 ± 0.9 practical salinity units (PSU) at the North Sea location and 11.3 ± 2.3 PSU at the Baltic sampling station (Figure S1, data from CMEMS https://doi.org/10.48670/moi‐00021). *Acartia tonsa* is only in the water column during the warm summer months (~June–October) and we therefore only included measurements from this timeframe. Live animals were sorted to species level and lab cultures started with approximately 200 adult *A. tonsa* individuals. Over days, cultures were gently adjusted to common‐garden conditions at 15.5°C (±0.3°C) and 15 PSU at a 12:12 light: dark regime. The microalgae *Rhodomonas* sp. were kept in the exponential growth phase at the same temperature and salinity as the cultures and fed at concentrations around 500 μgC/L every other day. Common‐garden conditions were maintained for at least three generations prior to the experiments. Full water changes were performed approximately every 3–4 weeks using filtered sea water (using a 0.2‐μm filter) and salinity was monitored and adjusted if necessary, using a mix of tap water and deionised water. All experiments were carried out at the culturing temperature of 15.5°C.

### 2.2 Physiological Assays

We conducted a series of experiments to assess the physiology and performance of both populations at low salinity conditions: acute mortality of adults, egg production and hatching, respiration and naupliar survival to adulthood. The data and statistical analysis for all experiments were done in R version 4.2.2 (R Core Team 2022).

#### 2.2.1 Acute Mortality

For the acute mortality experiment, 6 salinities were tested (0.1, 1, 2, 3, 5, and 15 PSU). To create the experimental salinities, filtered sea water was diluted with a mix of tap water and deionised water. In total, 90 recently matured females were picked, and five at a time were placed in one vial filled with 9 mL of water, so each salinity treatment included three independent replicates with a total of 15 individuals. During the duration of the experiment, no food was supplied and survival was monitored after 2 and 24 h by gently pipetting water up and down. Animals were scored as dead if they did not show any signs of movement to this stimulus. No individuals died at the 15 PSU control salinity. For the analysis, we fit a logistic regression and calculated the salinity at which 50% mortality (LS_50_) was observed for both populations using a generalised linear model with binomial distribution and the *dose.p* function from the *MASS* package (Venables and Ripley 2002).

#### 2.2.2 Egg Production and Hatching

To measure egg production, recently matured males and females were placed in pairs into 6‐well plates filled with 8 mL of filtered sea water at three salinities (5, 10 and 15 PSU) to mate and produce eggs. Each well contained one pair, with a total of six replicates per salinity. After 48 h, adults were removed. After another 48 h of hatching, the samples were fixed with a drop of 1% Lugol's solution. The contents were then moved to a petri dish with a counting grid, and all intact eggs and nauplii were counted. The number of total eggs laid consisted of all unhatched eggs plus the number of nauplii. Hatching success was defined as the number of nauplii divided by the number of total eggs produced.

#### 2.2.3 Respiration Rates

To determine base respiration rates, recently matured females were picked and acclimated to the experimental salinities for 16 h without food. Respiration was measured at two conditions, control (15 PSU) and low salinity (5 PSU). On the day of the experiment, animals were gently rinsed in filtered sea water and then placed in pairs into 80‐μL wells filled with filtered sea water at the experimental conditions. To ensure measurements were comparable, both populations were run at the same time and wells assigned at random. Oxygen concentrations were measured for up to 2 h with a PreSens optical sensor using the SDR_v4.0.0 software. Animals were preserved in 1% Lugol's solution and later photographed using a Nikon imaging microscope with NIS‐Elements software (v. 5.20.000). All pictures were taken with the same magnification to ensure consistency. Prosome length was then measured using the software ImageJ (Schneider et al. 2012). Respiration rates were calculated using the *respR* package (Harianto et al. 2019) and standardised per dry body weight using the length obtained from the images and the conversion factors as defined by Kiørboe et al. (1985). Since Lugol fixation is reported to reduce body size in copepods, we used the factors estimated by Jaspers and Carstensen (2009) to correct for shrinkage, though these effects should be consistent across all individuals.

To assess how acute exposure to extremely low salinity affects respiration, we conducted an additional experiment with even lower salt concentrations and a limited acclimation time of 2 h. Here, we measured the respiration rates of adult females at 2, 3, 5 and 10 PSU. As previous experiments showed that survival is not guaranteed with long‐term exposure (> 24 h) at these extremely low salinities below 5 PSU, we opted for a 2‐h acclimation period at the target salinity for this experiment. Afterwards, the procedure was identical to that described above in the first experiment.

#### 2.2.4 Survival to Adulthood

To test for the effect of low salinity on survival to adulthood, recently matured adults were placed in 100‐μm mesh cups inside beakers filled with 10 PSU water. Individuals were allowed to reproduce for 24 h after which the mesh cups containing the adult animals were removed, with the eggs falling through the mesh and remaining in the beaker. Another 24 h later, hatched nauplii were counted and placed in groups of 20 in 16 beakers (4 replicates per station and salinity) filled with 200 mL of filtered sea water at the experimental and control salinity (7 and 15 PSU, respectively). Previous experiments had shown high larval mortality at 5 PSU and therefore we chose 7 PSU as a low salinity condition in this experiment. In each beaker, *Rhodomonas* sp. was added at a concentration of 500 μgC/L and beakers were covered with aluminium foil to prevent evaporation. Survival was monitored after 3, 7, 10, 14 and 21 days by gently pouring the content of the beakers through a 50‐μm sieve and transferring the animals to a petri dish to count them under a stereo microscope. Survival and developmental stages were noted, distinguishing the following categories: nauplius, copepodite stage C 1–3, C 4–5 and C6 (adults). For adult individuals, the sex was noted. For the statistical analysis, we used a generalised linear mixed effect model (GLMM) accounting for repeated measurements using *lme4* (Bates et al. 2015).

### 2.3 Gene Expression Experiment

Because the cultures could be a mix of developmental stages, cultures were split to separate eggs from adults and the eggs were used to start a new, synchronised culture where growth was monitored to catch the onset of maturation. For each population, 15 replicates of 50 newly matured adults (750 total) were counted and transferred into cups with a 100‐μm mesh bottom for water exchange. The cups were inserted into three 20‐L acclimation tanks at culturing conditions (15 PSU, 500 μgC/L *Rhodomonas*), and animals were allowed to recover from handling overnight. Before the start of the experiment, a control (t0) was sampled from each tank by swiftly transferring the experimental cups into RNAlater; for this and all following treatments, there were three independent biological replicates. The remaining cups (12 for each population) were then placed into 7.5 L treatment tanks with a low salinity treatment (7 PSU) and a control treatment (15 PSU). Three hours post‐transfer, animals were sampled for the first time point (t1) by removing one cup per treatment and population from each of the three tanks. After 24 h, all remaining cups were sampled (t2). Each biological replicate (cup) was split into two 1.5‐mL tubes, each containing 25 animals and preserved in fresh RNAlater (full setup shown in Figure S2). Samples were stored at −20°C until RNA extraction.

### 2.4 RNA Extraction and Sequencing

RNA was extracted from pooled animals (*N* = 25 per tube) using TRIzol reagent. For sample purification, Qiagen RNeasy spin columns were used, adding a DNAse treatment and second buffer RPE wash to improve sample purity. Sample quality and concentration were assessed using Qubit Fluorometer (Invitrogen) and Nanodrop (Thermo Fisher). Samples were stored at −80°C before sequencing. In cases where RNA was extracted from both tubes of a given biological replicate, the higher‐quality sample was selected for sequencing. A total of 30 samples (three biological replicates per treatment, population and time point) were used for the gene expression analysis. Library preparation and sequencing were performed by Novogene UK on an Illumina Novaseq X (see Table S8 for sample overview).

Raw reads were quality assessed using *FastQC* (Andrews 2010) and *MultiQC* (Ewels et al. 2016), adapters were trimmed using *fastp* (Chen et al. 2018). We then used *salmon* (Patro et al. 2017) to map reads to an existing *Acartia tonsa* transcriptome with ENA accession HAGX01 (Jørgensen et al. 2019). Mapping rate was 73.65% ± 0.37% (see Table S7). After filtering out low counts, 25,567 transcripts were included in the downstream analysis, 13,872 of which had a functional annotation.

### 2.5 Transcriptomic Analysis

The differential gene expression analysis was done in R v. 4.2.2. utilising the *DESeq2* package (Love et al. 2014). We visualised variance stabilised transformed and regularised log transformed data in PCA plots highlighting time and treatment for both stations (Figure S6). We employed two different approaches to analyse differential gene expression. First, we used four separate likelihood‐ratio‐tests (LRTs) to extract transcripts that (1) were significantly differentially expressed at low salinity, (2) showed a time‐dependent expression pattern at low salinity, (3) showed a population‐dependent expression pattern at low salinity and (4) showed a population and time‐dependent expression pattern at low salinity. Due to the imbalanced model design with no treatment applied at the control time point 0, it was necessary to manually edit the model matrices used as input for the LRT models in accordance with the *DESeq* manual. Second, to get more detailed insight into genes that had a time‐dependent salinity expression, we collapsed descriptors into an interaction term and used pairwise comparisons to contrast treatment and time point combinations for both stations separately. In all cases, transcripts were included in the final gene set if adjusted *p*‐values were lower than 0.05 using the Benjamini‐Hochberg false discovery rate as correction. Genes were considered over‐expressed if log_2_fold changes were > 0 and under‐expressed if log_2_fold changes were < 0. Differentially expressed genes (DEGs) were normalised and visualised using the *pheatmap* package (Kolde 2019). To visualise patterns of expression over time, we used the *degPatterns* function from *DEGreport* (Pantano 2022), grouping DEGs with similar expression patterns and requiring a minimum of 10 genes per valid cluster.

To test for functional enrichment in the target gene sets, we used over‐representation analysis (ORA) and gene set enrichment analysis (GSEA) with gene ontology (GO) from *clusterProfiler* (Wu et al. 2021). Only genes that remained after filtering were included in the gene background against which the significant sets were tested for GO enrichment, and an adjusted *p*‐value cutoff of 0.05 was used (using the Benjamini–Hochberg false discovery rate correction). For the gene sets identified with pairwise comparisons, we used the *compareCluster* function of *clusterProfiler* and visualised the enriched GO terms with the *dotplot* function (Wu et al. 2021).

## 3 Results

### 3.1 Physiological Assays

Physiological assays revealed both conserved and divergent plastic responses to acute salinity challenge. Salinity had an impact on survival for both populations, where survival was significantly lower at low salinities (*p* < 0.001) and decreased from 2 h to 24 h (*p* = 0.003; Figure 1C). However, North Sea copepods had lower survival at both timepoints (population effect; *p* = 0.012), and a significant salinity and population interaction indicated that Baltic Sea copepods were able to tolerate and survive in lower salinities to a greater degree than North Sea copepods (*p* = 0.004, LS_50_ after 2 h: 0.76 ± 0.25 (Baltic), 1.10 ± 0.16 (North), and 24 h: 1.34 ± 0.52 (Baltic), 2.46 ± 0.19 (North); Figure 1B,C). Oxygen consumption of acclimated individuals did not differ in their response to salinity challenge. After a 16‐h acclimation period at 5 and 15 PSU, respiration rates were significantly different between stations (*p* < 0.001, Figure S3), but not treatment salinity (*p* = 0.844). Baltic organisms had higher oxygen consumption at both treatment and control conditions compared to North Sea copepods: 5 PSU: 0.387 ± 0.132 (Baltic), 0.192 ± 0.064 (North), 15 PSU: 0.392 ± 0.079 (Baltic), 0.174 ± 0.046 (North), all measurements in μmol O_2_ h^−1^ mg dry body weight^−1^. In contrast, when challenged acutely (2‐h acclimation period), salinity had a significant effect on respiration rates (*p* < 0.001) but not station (*p* = 0.701) with respiration rates decreasing with decreasing salinity (Figure 1D; 5 PSU: 0.555 ± 0.104 (Baltic), 0.684 ± 0.194 (North), 2 PSU: 0.431 ± 0.065 (Baltic), 0.466 ± 0.141 (North)).

Overall, acutely challenged animals had higher respiration rates compared to the measurements after the 16‐h acclimation. This observed effect of acclimation time on oxygen consumption can likely be explained by the higher metabolism of non‐starved animals during the 2‐h acute acclimation (Kiørboe et al. 1985).

Nauplii survival was also significantly influenced by treatment (*p* < 0.001) and time (*p* < 0.001). There was a significant effect of population on survival, with Baltic copepods showing higher survival (*p* = 0.013). However, the low treatment salinity of 7 PSU was lethal for nauplii from both populations, where no individual reached the copepodite stages and no individuals survived beyond 14 days (Figure S4). At the control salinity, adults were recorded after 14 days, with all surviving individuals reaching adulthood after 21 days (North Sea: 42.5% ± 6.5%, Baltic Sea: 50.0% ± 10.0%). The female: male ratio differed drastically between populations (2.08 for Baltic Sea, 0.28 for North Sea).

Lastly, neither egg production nor hatching was significantly influenced by salinity (*p* = 0.737 and *p* = 0.517, respectively) or population (*p* = 0.281 and *p* = 0.658, respectively). Daily egg production was highly variable and averaged at 34.3 ± 25.8 for the North Sea population and 26.8 ± 14.0 for the Baltic population across all treatments. For the North Sea population, 20.8 ± 15.9 nauplii hatched (60.7% of all eggs), and for the Baltic Sea population, 18.8 ± 10.3 nauplii hatched (70.3% of all eggs; Figure S5).

### 3.2 Gene Expression

We first identified genes that showed a significant response to low salinity using the LRT model. We found 889 DEGs for which gene ontology (GO) enrichment yielded 8 terms that were significantly enriched in both ORA and GSEA. Here, the biological processes were exclusively related to transport functions (GO:0055085 transmembrane transport, GO:0006811 ion transport, GO:0006814 sodium ion transport, see Table S1). Of these DEGs, 105 were significant for an interaction between low salinity and time, with enrichment for 18 GO terms, 7 of which were biological processes. Again, functional enrichment of biological processes was dominated by transport processes (GO:0055085 transmembrane transport, GO:0006811 ion transport, GO:0006814 sodium ion transport, GO:0006869 lipid transport, see Table S2).

**FIGURE 2:**
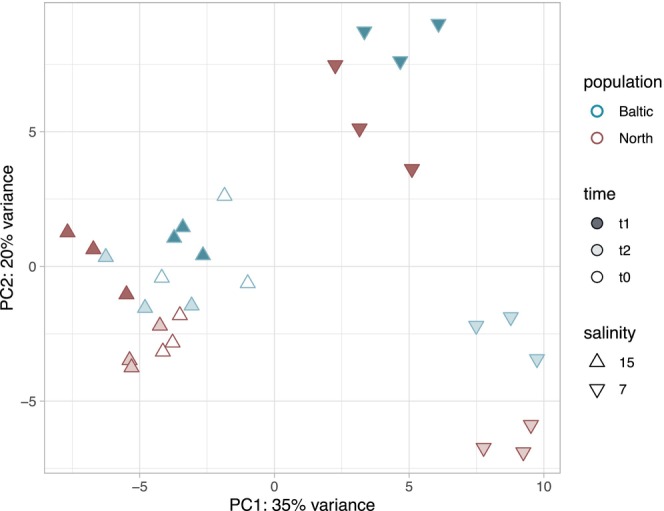
PCA of 889 genes significantly differentially expressed at low salinity (likelihood ratio test). Samples from the Baltic are shown as blue triangles and the North Sea as red triangles. Treatment salinity was measured in practical salinity units (PSUs), t0 is the control sampling before transfer to the treatment salinities, and t1 and t2 were sampled 3 and 24 h post transfer, respectively.

Though the LRT only identified two genes as differentially expressed for the two‐way interaction between low salinity and population, and one for the three‐way interaction between low salinity, population and time, the PCA of the 889 significant salinity transcripts shows clear clustering by both time and population (Figure 2). We therefore opted for pairwise comparisons to gain further insights into more subtle population differences in contrast to the broad patterns identified by the LRT (Love et al. 2014).

When using pairwise comparisons, contrasting the low salinity treatment for each station and time point with the respective controls, 341 DEGs were identified at low salinity for either t1 or t2 in the Baltic population, and 415 DEGs for the North Sea copepods, totalling 602 unique salinity‐dependent genes identified by pairwise comparisons. Of these, 516 DEGs were shared with the salinity‐dependent LRT (85.7% of all pairwise transcripts and 58.0% of all LRT transcripts, Table S4).

#### 3.2.1 Conserved Salinity Response

**FIGURE 3:**
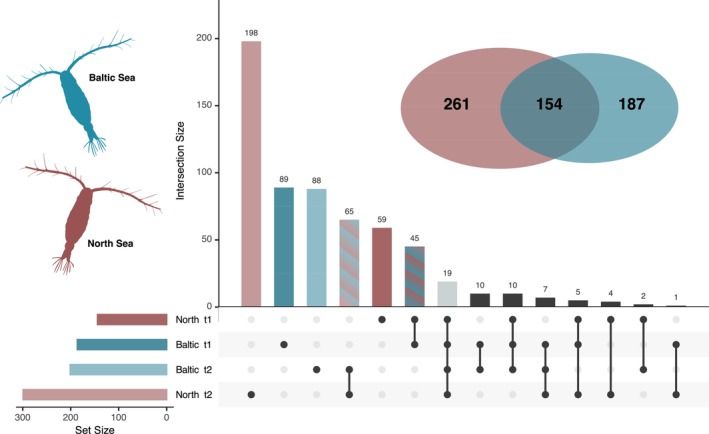
Counts of significantly expressed genes for Baltic (labelled in blue) and North Sea (labelled in red) *Acartia tonsa* exposed to low‐salinity stress for 3 h (t1) and 24 h (t2) identified with pairwise comparisons. The Venn diagram shows the overall significant genes at both time points as well as the overlap between populations. The upset plot shows unique and intersecting genes between populations and time.

Across both populations, 154 pairwise genes were jointly differentially expressed at low salinity (Figure 3), of which 63 were also identified by the time‐and salinity‐dependent LRT. This conserved plastic response to salinity included 79 transcripts for the short‐term response (t1, 3 h) and 91 transcripts for the long‐term response (t2, 24 h), highlighting time‐dependent shifts in gene regulation.

**FIGURE 4:**
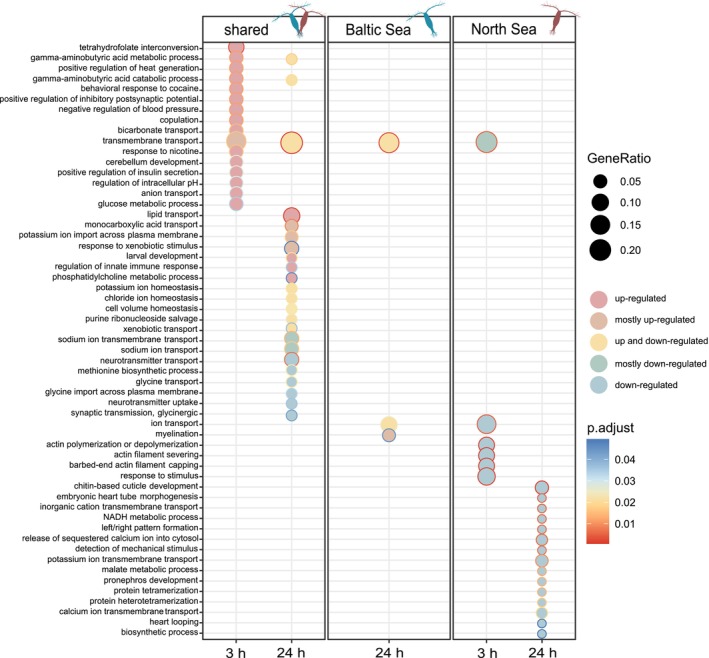
Gene ontology enrichment for biological processes (BPs) of time‐dependent salinity genes for shared and unique genes of Baltic and North Sea *Acartia tonsa* . Enrichment was calculated with the *compareCluster* function of *clusterProfiler* (v 4.0, Wu et al. 2021). Regulation of genes within each GO term is indicated by fill; outline colour indicates adjusted *p*‐values (using the Benjamini–Hochberg false discovery rate correction).

GO enrichment revealed time‐dependent patterns of biological processes in response to low salinity exposure. In the short‐term response, functional enrichment was primarily driven by upregulated genes, with key processes related to osmoregulation and ion transport (GO:0055085 transmembrane transport, GO:0015701 bicarbonate transport, GO:0006820 anion transport). Additionally, metabolic and neurological regulation was enriched, including GABA metabolism (GO:0009450 and GO:0009448) and glucose metabolic process (GO:0006006). Other enriched functions suggested a systemic response to salinity stress, such as positive regulation of heat generation (GO:0031652), modulation of inhibitory postsynaptic potential (GO:0097151) and behavioural responses (GO:0048148 behavioural response to cocaine; Figure 4, full list in Table S3; molecular functions: Table S5, cellular components: Table S6). Several DEGs associated with osmoregulatory processes were identified, including sodium‐dependent glucose transporters (*G5CMC2* and *RPPX_22905*), a major facilitator superfamily (MFS) type transporter (*SLC18B1*) and carbonic anhydrase (*CAA6C*), suggesting active ion regulation (full list in Table S4).

After 24 h of exposure, transmembrane transport remained a key enriched function, but transport‐related processes diversified, with both upregulated and downregulated transport pathways emerging. Upregulated genes were associated with lipid transport (GO:0006869), monocarboxylate transport (GO:0015718) and potassium ion import (GO:1990573), while downregulated genes drove enrichment in sodium ion transmembrane transport (GO:0035725) and glycine transport (GO:0015816). The prolonged stress response was also characterised by enrichment in stress response mechanisms, including response to xenobiotic stimulus (GO:0009410) and regulation of innate immune response (GO:0045088). Additionally, key homeostatic processes were enriched, such as potassium ion homeostasis (GO:0055075), chloride ion homeostasis (GO:0055064) and cell volume homeostasis (GO:0006884), indicating a shift towards long‐term osmoregulatory adjustments. Notably, genes associated with these enriched processes included a solute carrier (*SLC12A3*), an organic cation transporter (*CGI_10003241*) and a carbonic anhydrase (*CA4‐like*), further supporting the role of active ion regulation in coping with salinity stress.

#### 3.2.2 Population‐Specific Response

A total of 187 and 261 genes showed unique differential expression in response to low salinity for the Baltic and North Sea populations, respectively (Figure 3). For the Baltic population, 99 DEGs (89 unique to t1; 10 shared between t1 and t2) were differentially expressed during short‐term exposure (t1), while 98 DEGs (88 unique to t2; 10 shared between t1 and t2) were differentially expressed after 24 h (t2). No biological functions were significantly enriched at t1. However, by t2, upregulated genes predominantly contributed to enrichment in three key biological processes: Transmembrane transport (GO:0055085), ion transport (GO:0006811) and myelination (GO:0042552) (Figure 4). Among the differentially expressed osmoregulatory genes, cation transporting ATPases (*ATP13A3* present at both time points), organic cation transporters (*R4UKP6, OCT1*) and an MFS transporter (*Orct_13*) were identified.

In contrast, the North Sea population exhibited a more extensive transcriptional response over time with 63 DEGs (59 unique to t1; 4 shared at t1 and t2) identified for the short‐term response, increasing to 202 DEGs (198 unique to t2 + 4 shared at t1 and t2) at t2. At both time points, functional enrichment was driven by downregulated genes, suggesting a suppression of key biological processes. At t1, downregulated functions were associated with structural reorganisation, including actin filament severing (GO:0051014) and actin filament capping (GO:0051016), alongside suppressed ion and transmembrane transport processes (GO:0006811, GO:0055085) (Figure 4). Notably, osmoregulatory genes downregulated at this stage included receptor potential cation channels (*TRPA‐1*). By t2, suppression extended beyond transport processes to include biosynthesis and metabolism (GO:0009058 biosynthetic process, GO:0006108 malate metabolic process, GO:0006734 NADH metabolic process), developmental pathways (GO:0048793 pronephros development, GO:0008362 chitin‐based cuticle development) and ion homeostasis mechanisms (GO:0098662 inorganic cation transmembrane transport, GO:0071805 potassium ion transmembrane transport, GO:0070588 calcium ion transmembrane transport). Among the significantly downregulated osmoregulatory genes at this stage were voltage‐gated potassium channels (*KCNV1*, *AMK59_7147*).

**FIGURE 5:**
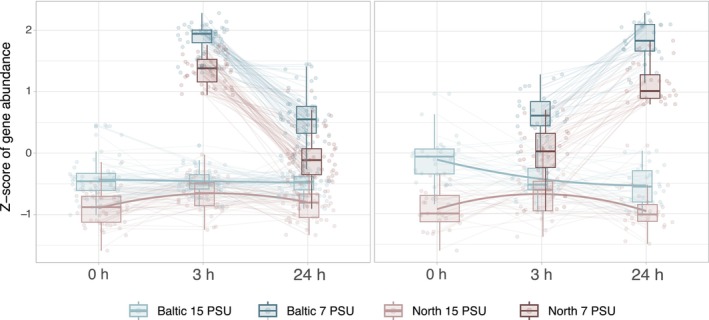
Gene clusters with similar expression patterns at low salinity show population differences in expression levels. Here the two strongest clusters (most DEGs) are shown. Left: 48 of 154 shared genes are differentially expressed at low salinity treatment; Right: 27 of 154 shared genes are differentially expressed at low salinity treatment. Clustering performed using *degPatterns* from *DEGreport* (Pantano 2022).

When comparing relative expression patterns, genes differentially expressed at low salinity generally exhibited higher expression levels in the Baltic population (Figure 5). While the overall direction of gene expression changes was consistent between populations, the North Sea population displayed relatively lower expression levels at low salinity, particularly after 24 h (Figures S8 and S9).

## 4 Discussion

We used physiological experiments on copepods common‐gardened for multiple generations combined with a transcriptomic assay to investigate local adaptation to salinity in a Baltic and North Sea population. If local adaptation to low salinity was present, we expected to see higher fitness in the low salinity Baltic Sea copepods than those originating from the high salinity North Sea when exposed to low salinity stress. The increased survival of the Baltic Sea individuals at low salinity relative to the North Sea population is indicative of local adaptation to salinity. However, not all phenotypic measures supported this conclusion as there were no consistent differences in metabolism and reproduction. The gene expression responses allowed us to identify the conserved plastic response to low salinity stress that enables *A. tonsa* as a species to tolerate variable osmotic environments and, as might be expected, included functions related to ion transport and homeostasis (Figure 4). Population‐level differences in expression were primarily driven by increased differential expression in the high salinity North Sea population, suggesting an inability to efficiently osmoregulate and tolerate low salinity conditions relative to the Baltic Sea population. Our findings suggest local adaptation to the low salinity conditions in the Baltic Sea with an increased tolerance to these dilute conditions but also highlight the extraordinary plasticity of *A. tonsa* as a species across broad osmotic environments.

### 4.1 Phenotypic Traits Reveal Local Adaptation

Baltic Sea and North Sea populations showed phenotypic differences in response to low salinity challenges that were consistent with local adaptation. As expected, extreme low salinity increased mortality in both populations, but Baltic Sea copepods showed significantly higher survival than their North Sea counterparts. Despite this evidence for local adaptation in short‐term survival, other physiological and reproductive traits did not differ between populations. Oxygen consumption rates were similarly affected by low salinity in both populations, and reproductive success—measured by egg production and hatching rates—remained unchanged.

Typically, *A. tonsa* exhibits an increase in respiration rates when exposed to salinities deviating from its native environment (Gaudy et al. 2000; Lance 1965), but salinities below 5 PSU have not been considered in past experiments. In extremely low salinity environments, copepods reduce their swimming activity (Seuront 2006), which we anecdotally observed in our low salinity treatments. This behavioural response could explain why, despite experiencing osmotic stress, overall metabolic rates were reduced.

Our experiments simulated an acute salinity shock, mirroring natural salinity reductions during heavy rain falls or tidal cycles. In these cases, evolving a short‐term strategy to tolerate extreme hyposaline conditions is necessary to ensure survival. Prolonged exposure could be avoided by behavioural responses such as migrating to deeper, more saline layers. Indeed, decreased surface salinity alters the vertical migration patterns of copepods (Lance 1962) likely to avoid prolonged exposure to harmful conditions. Our experimental setup enabled us to uncover population differences in survival but not reproductive success since the latter trait might not be impacted by short‐term exposure of adult individuals to low salinity. This is especially true since parental investments in egg production likely have been made prior to the start of the experiments (Niehoff 2007). In similar experiments on the reproduction of *Acartia* with longer acclimation periods, egg production rate decreased at low salinities (Dutz and Christensen 2018; Peck and Holste 2006).

The observed difference in female: male ratios between populations during the naupliar survival experiment should be taken cautiously. Anecdotal observations from maintaining the cultures under controlled salinities indicate that sex ratios are generally similar across populations. Instead, the discrepancy is likely explained by a combination of high natural variation in sex ratios in *A. tonsa* clutches, where individual females can produce clutches ranging from strongly female‐ to male‐biased (Burris and Dam 2015), and the relatively small sample size used in this part of the experiment.

While short‐term responses appear critical for immediate survival, the long‐term strategy to tolerating the overall reduction in salinity in the Baltic Sea may include more subtle phenotypes that would only emerge under long‐term acclimation or development at low salinities. We initially planned to assess reproductive output in copepods reared at low salinity, but complete naupliar mortality at these conditions (Figure S2) prevented further experiments. This suggests that, at least in the populations used in this study, there may be a lower limit to population persistence under salinity decreases and that developmental stages differ in their salinity tolerance (Magouz et al. 2021; Nour et al. 2021). Future studies should quantify the long‐term effects of low salinity exposure on fitness in *A. tonsa* from a suite of osmotic environments.

### 4.2 Core Mechanisms of Osmoregulation in *A. tonsa*

Typically, most invertebrates are isoosmotic, meaning their internal osmolarity matches their surroundings. For isoosmotic organisms, a decrease in environmental salinity leads to an inflow of water into the cells that is initially counteracted by the release of inorganic ions such as K^+^, which reduces internal osmolarity and helps prevent further swelling (Moran and Pierce 1984). Since ion concentrations are important for cell functions due to their role in maintaining membrane potentials, over intermediate timeframes, compatible organic osmolytes such as free amino acids (FAA) are used to maintain isoosmotic conditions (Otto and Pierce 1981; Yancey 2005). When stressful conditions persist for longer time periods or when salinity reaches a critical lower limit, organisms are forced to engage in energetically costly active ion regulation to sustain cell function (Lee et al. 2011), and as a consequence, organisms become hyperosmotic compared to their surroundings (Mantel and Farmer 1983).

The osmoregulatory strategy of *A. tonsa* has not been extensively investigated. *Acartia tonsa* has been found to be a weak hyperosmotic osmoregulator, maintaining isoosmotic body fluids at native salinities and becoming slightly hyperosmotic when salinity decreases (Lance 1965; Mantel and Farmer 1983). There is evidence for osmoregulatory efforts such as regulation of ion concentrations and amino acid levels to maintain constant cell volumes in low salinity (Farmer and Reeve 1978; Svetlichny and Hubareva 2014). This is consistent with the osmoregulatory mechanism of other copepods. For example, both the cyclopoid copepod *Apocyclops royi* (Jepsen et al. 2025) and *Eurytemora affinis* are isoosmotic at higher salinities, but osmoregulate when exposed to low salinity (Gerber et al. 2016; Johnson et al. 2014).

Our findings indicate that *A. tonsa* likely follows the standard model of invertebrate osmoregulation, showing both short‐term regulation of ions as well as intermediate regulation of amino acids to maintain homeostasis. The large core set of genes that consistently responded to the low salinity challenge between Baltic and North Sea populations illuminates how *A. tonsa* is able to tolerate considerable salinity decreases.

Under low salinity stress, it is necessary to control both intercellular ion homeostasis and cell volume, which require active transmembrane transport and water balance, which are energetically costly and can affect intracellular pH levels (Krasznai et al. 1995; Kültz 2015). Accordingly, the immediate response to low salinity was dominated by metabolic upregulation, as well as ion transport and pH regulation in an initial effort to combat salt stress and maintain homeostasis.

With prolonged exposure to low salinity, the functional response broadened beyond ion transport to include the transport of larger molecules such as amino acids and lipids, indicating a shift in osmoregulatory mechanisms. Glycine, in particular, plays a key role as an organic osmolyte in many marine invertebrates under hypo‐osmotic stress (Farmer and Reeve 1978; Podbielski et al. 2022). Farmer and Reeve (1978) identified glycine, serine and proline as important free amino acids for osmoregulation in *A. tonsa* , all of which they found in reduced concentrations following exposure to low salinity. Consistent with this, we show that glycine transport and glycinergic synaptic transmission are downregulated after 24 h, likely reflecting adjustments in gene expression in response to declining FAA levels. The regulation of lipid transport may support plasma membrane integrity during persistent osmotic stress (Mu et al. 2024). The activation and regulation of diverse transport functions in response to low‐salinity stress have been observed across multiple marine taxa, including euryhaline fish, oysters and copepods (DeBiasse et al. 2018; Jones et al. 2019; Li et al. 2025).

In many crustaceans, Na^+^/K^+^‐ATPase and V,H^+^‐ATPase (VHA) are important osmoregulatory genes (Gerber et al. 2016; Lee et al. 2011; Lv et al. 2013; Posavi et al. 2020), and we expected to observe these candidate genes in the core response to salinity in *A. tonsa* . While these specific genes were not differentially expressed in our samples, we found a suite of ion transporters responding to salinity challenges, including Na^+^ and Cl^−^ dependent transporters. It is likely that these transporters are serving a similar role in contributing to osmoregulation. For example, at t1 and t2, both populations showed upregulation of the sodium chloride cotransporter (*NCC*; *SLC12A3*) that reabsorbs Na^+^ and Cl^−^ in the urinary bladder of teleost fishes exposed to freshwater (Takvam et al. 2021). Alternatively, fully assembled ATPases could be stored in vesicles and be activated and translocated upon stress, as has been shown with VHA in sharks (Tresguerres et al. 2010). This would allow animals to quickly respond to salinity changes without activating transcriptional pathways.

Further, we found *carbonic anhydrases* which are linked to osmoregulation in crustaceans and previously were shown to be upregulated under low salinity stress in the Pacific white shrimp (Roy et al. 2007). Carbonic anhydrases convert respiratory CO_2_ to bicarbonate and protons to take up ions such as Na^+^ and Cl^−^ via, for example, anion exchangers, VHA and sodium proton exchangers (NHE); carbonic anhydrases are likely core to the copepod osmoregulatory strategy (Lee 2023; Lee, Charmantier, et al. 2022). In addition, both populations upregulated genes in the GABA pathway. The regulation of GABA pathways plays an important role in salinity tolerance, as this neurotransmitter can increase tolerance to abiotic stress by maintaining membrane potential and promoting ion transport and has been found to accumulate in response to salt stress in plants (Cheng et al. 2018; Su et al. 2019; Yuan et al. 2023). Further, *major superfacilitator family* (MFS) genes were differentially expressed in both populations. MFS genes are a family of transporters operating under a chemiosmotic ion gradient and include sugar transporters (Pao et al. 1998) such as the *sodium‐dependent glucose transporter 1* that was upregulated at t1. Finally, De Vos et al. (2019) identified several groups of transport genes that were differentially expressed in *Artemia* brine shrimp following salt stress, such as ion transporters and salt‐dependent transporters. Similarly, we found various solute carrier genes including *organic cation transporter‐like* or *organic anion transporter family member 2A 1*. Similarly, carbonic anhydrases, and a suite of ion transporters were differentially expressed in the copepod *E. affinis* under salinity stress (Posavi et al. 2020), indicating that these genes or their functional equivalents are important for osmoregulation across diverse copepods.

The time‐sensitive pattern of the stress response we observed suggests a dynamic osmoregulatory strategy common to invertebrates. Initially, *A. tonsa* seems to rely mainly on active ion transport to counteract the immediate osmotic shock, regulating intracellular ion concentrations and pH levels. However, after prolonged exposure, the shift towards organic osmolytes and lipids may indicate a transition to a more stable state, potentially reducing the high energetic demands of active osmoregulation and avoiding depletion of essential ions. Future studies should investigate if *A. tonsa* can reach a fully isoosmotic state at the salinity we tested or if continued exposure to low salinity will force the organism to maintain hyperosmotic levels through active osmoregulation.

### 4.3 Population‐Specific Gene Expression Underlying Local Adaptation

We identified three main differences in gene expression that may underlie local adaptation between the populations. First, more genes with a time‐dependent salinity response were expressed in the North Sea population. From 3 h to 24 h, this population more than tripled the number of population‐specific DEGs, while the number of population‐specific DEGs in the Baltic Sea population remained stable (Figure 3). The lower numbers of DEGs may reflect increased resilience of the Baltic Sea population to low salinity stress, as they are able to recover and maintain homeostasis more efficiently, similar to transcriptomic resilience (Franssen et al. 2011). The European flounder (Larsen et al. 2007) and the copepod *Tigriopus californicus* (DeBiasse et al. 2018) similarly have muted transcriptomic responses that correspond to higher salinity tolerance, plasticity and local adaptation. In addition, the North Sea DEGs were almost exclusively downregulated, meaning that functions were being suppressed. This suggests that the North Sea copepods were prioritising survival over growth‐limiting metabolic demands and costly active ion transport, especially after prolonged exposure to low salinity (Hand and Hardewig 1996).

Second, many of the genes associated with low salinity responses in both populations were more highly expressed in the Baltic population during the stressful condition, and in some cases also the control state. This is similar to the phenomenon known as ‘front‐loading’ that has been described first in corals where resilient corals showed higher expression levels of important stress‐related genes in an undisturbed state (Barshis et al. 2013). We suggest that the Baltic population could have adapted to low salinity conditions by evolving an increased basal expression of key osmoregulatory genes, allowing them to more efficiently respond to sudden decreases in salinity.

Lastly, some genes, which have been linked to osmoregulation in crustaceans, were uniquely differentially expressed only in a single population. North Sea copepods showed differential expression of *arginine kinase*, an enzyme that facilitates ATP formation by transferring phosphate from phospho‐L‐arginine, the phosphagen ATP buffer in many invertebrates (Holt and Kinsey 2002). In the blue crab *Callinectes sapidus*, hypo‐osmotic conditions induced a higher arginine kinase flux, and in the estuarine copepod *E. affinis* , arginine kinase was identified as a key osmoregulatory gene in response to fresh water exposure (Holt and Kinsey 2002; Posavi et al. 2020). Only Baltic copepods showed significant regulation of *ATPase* genes (e.g., *ATP13A3, P‐type ATPase*), which use ATP as an energy source to fuel the active exchange of ions across the cell membrane. These differences in expression of osmoregulatory genes could point towards adaptation of the Baltic population to the decreased salt levels in their natural habitat and explain the higher survival we observed in this study.

## 5 Conclusions and Implications for Future Changes

We showed that *A. tonsa* as a species is able to withstand extreme salinity conditions for hours to days. Given these findings, we assume that *A. tonsa* will be able to tolerate climate‐driven salinity declines in most of its distribution, the exception being the north‐east Baltic Sea. Since this low salinity area represents the outermost limit of the current species' distribution, further decreases in surface salinity might lead to unfavourable conditions for larval development and therefore range contractions.

Despite their broad osmotic tolerance, we found evidence for local adaptation to low salinity, indicating that *A. tonsa* from higher salinity regions can adapt and thrive under decreased salinity regimes. The relatively rapid adaptation to low salinity observed here has been driven by an expansion of salinity tolerance, consistent with other organisms moving from high to low salinity (Brennan et al. 2015; Lee et al. 2011). The tolerance to low salinity appears to be driven by a more efficient physiological response (Figure 2) that includes front‐loading of key osmoregulatory genes (Figure 5). This is especially remarkable since *A. tonsa* might not be native to the Baltic Sea and was first recorded there around 100 years ago (Smirnov 1935), but has rapidly established a presence as a dominant Acartia species in the summer months. It is possible that this establishment was facilitated via ship ballast water and could have contributed to the movement of adaptive alleles between populations. However, it remains unclear if the early 1900s truly represent the first appearance of *A. tonsa* in European waters (Redeke 1934). Even if the species was present in the Baltic Sea and simply not identified prior to the 1900s, the Baltic Sea—previously a landlocked lake—only gained a connection to the North Sea around 8000 years ago (Björck 1995). This marks the earliest point in time where a marine species such as *A. tonsa* could have colonised and adapted to the low salinity Baltic.

The evidence for local adaptation presented here indicates that *A. tonsa* has the capacity to evolve tolerance to low salinities and—in the past—has adapted to novel environments. This suggests that the species potentially can adapt to future changes in ocean salinity following the space‐for‐time concept (Kharouba and Williams 2024). Its high genetic variation and rapid generation times have enabled it to adapt to other contemporary anthropogenic changes over just tens of generations (Brennan et al. 2022a), and this adaptive capacity could also extend to future changes in salinity. However, rapid evolution can have trade‐offs such as loss of plasticity or reduced fecundity (deMayo et al. 2025; Brennan et al. 2022b). Indeed, habitat shifts and local adaptation from high to low salinity in other organisms have been accompanied by expansions of plasticity to tolerate low salinity, but a loss of plasticity to tolerate the ancestral environment (DeFaveri and Merila 2014; Marchinko and Schluter 2007) consistent with theoretical predictions of genetic assimilation (Lande 2009). If *A. tonsa* originating from low salinity carries the plasticity to tolerate both low and high salinity fluctuations is currently not known, but adaptation to low salinity could carry costs (Sasaki and Dam 2021). Future studies should address the limits of *A. tonsa'*s adaptive potential, the impacts of rapid adaptation on plasticity and investigate potential trade‐offs of rapid adaptation to low salinity.

## Author Contributions

A.H. and R.S.B. designed this study. A.H. and R.S.B. collected the animals. G.A., J.C.N.‐S. and A.H. took care of animal cultures. A.H. conducted the experiments with assistance from J.C.N.‐S., G.A. and R.S.B. A.H. processed and analysed the data with input from T.B. and R.S.B. A.H. and R.S.B. wrote the manuscript. All authors edited and contributed to the final version of the manuscript.

## Funding

This work was supported by Deutsche Forschungsgemeinschaft (504958481).

## Conflicts of Interest

The authors declare no conflicts of interest.

## Supporting information


**Figure S1:** Daily surface salinity measurements for both sampling locations from 1993 to 2021, includes all measurements from June to October. Coloured points indicate mean salinity values for the Baltic location (blue) and North Sea location (red). Data from CMEMS https://doi.org/10.48670/moi‐00021.
**Figure S2:** (A) Schematic of experimental setup. Coloured circles indicate experimental cups each containing 50 adult animals with three biological replicates for each time point and treatment (roman numerals I—III). Cups were placed and sampled at random, and the order in this schematic does not reflect the actual experiment. Experimental animals in each cup were divided into two pseudo‐replicates with only one of them being forwarded for RNAseq and one as back‐up in case of unsuccessful extractions. (B) Picture of the acclimation tanks, Baltic samples are marked with green tape. (C) Experimental tank after sampling t1; 7 PSU treatment is marked in blue, 15 PSU treatment is marked in black.
**Figure S3:** Respiration rates of *Acartia tonsa* individuals from the Baltic Sea (blue) and the North Sea (red), standardised per dry body weight. Measurements were conducted after 16 h of acclimation to the treatment salinities.
**Figure S4:** Naupliar survival of *Acartia tonsa* from the Baltic (blue) and North Sea (red) at two treatment salinities (7 and 15 PSU); points indicate mean survival at sampling days, error bars indicate standard deviations.
**Figure S5:** Egg production (top panel) and hatching success (bottom panel) of *Acartia tonsa* from the Baltic (blue) and North Sea (red) after acute transfer to three treatment salinities (5, 10, 15 PSU).
**Figure S6:** PCA of all 25,567 genes that remained passed filtering; the Baltic population is coloured in blue, the North Sea population in red. Left: ellipses show clustering by population and time; right: ellipses show clustering by population and treatment.
**Figure S7:** Differentially expressed genes that were shared between North and Baltic Sea (154 DEGs) clustered by expression patterns (minimum gene count per cluster = 10), clustering done using *degPatterns* from *DEGreport* (Pantano 2022), Baltic Sea shown in blue, North Sea in red.
**Figure S8:** Salinity genes that were differentially expressed in North Sea and/or Baltic Sea samples (602 DEGs, including the 154 shared DEGs shown in Figure S7) show patterns in relative expression (minimum gene count per cluster = 10), clustering done using *degPatterns* from *DEGreport* (Pantano 2022), Baltic Sea shown in blue, North Sea in red.
**Figure S9:** Differentially expressed salinity genes identified by pairwise comparisons in *DESeq2* (Love et al. 2014). Over‐expressed genes have positive values assigned (log_2_ fold change > 0, dark colours), and under‐expressed genes have negative values assigned (log_2_ fold changes < 0, light colours). Population‐specific DEGs for the Baltic Sea are shown in blue, for the North Sea in red, and DEGs shared between both populations in grey.
**Figure S10:** Phylogenetic tree of genotyped North and Baltic Sea individuals mapped to sequences of known species and clade identity. Mapping of mtCOI sequences followed a Bayesian approach. Baltic samples are shown in blue, North Sea samples in red.

**Table S1:** Gene ontology enrichment with GSEA and ORA for DEGs with a salinity effect, identified with an LRT model, ontology abbreviations MF, molecular functions; BP, biological processes; CC, cellular components.
**Table S2:** Gene ontology enrichment with GSEA and ORA for DEGs with a salinity and time interaction, identified with an LRT model, ontology abbreviations MF, molecular functions, BP, biological processes, CC, cellular components.
**Table S3:** Gene ontology enrichment for biological processes (BP) with ORA for pairwise gene sets. Separate enrichment analyses were conducted per time point (t1 and t2) and gene set (both = present in North and Baltic DEGs, SW08 = Baltic Sea, WH = North Sea).
**Table S4:** List of annotated differentially expressed salinity transcripts, identified with LRT or pairwise comparison. Presence of a transcript is indicated with 1, absence with 0. Log2fold changes are supplied for pairwise comparisons.
**Table S5:** Gene ontology enrichment for molecular functions (MF) with ORA for pairwise gene sets. Separate enrichment analyses were conducted per time point (t1 and t2) and gene set (both = present in North and Baltic DEGs, SW08 = Baltic Sea, WH = North Sea).
**Table S6:** Gene ontology enrichment for cellular components (CC) with ORA for pairwise gene sets. Separate enrichment analyses were conducted per time point (t1 and t2) and gene set (both = present in North and Baltic DEGs, SW08 = Baltic Sea, WH = North Sea).
**Table S7:** Sample info for post QC reads, mapping rates are given in %.
**Table S8:** Metadata and sample accessions of all RNA samples used in the study and stored in bioproject PRJNA1258960 on NCBI.

## Data Availability

The code and physiological data that support the findings of this study are archived and openly available at Zenodo (http://doi.org/10.5281/zenodo.15535306). Transcriptomic read data and sample metadata are available at NCBI under the project accession PRJNA1258960.
