# Supplementary material for: Local Adaptation and Osmoregulatory Mechanisms of the Copepod Acartia tonsa Under Low Salinity Stress
Source: Mol Ecol. 2025 Dec 14;35(1):e70208. doi: 10.1111/mec.70208 (PMC12745923; doi:10.1111/mec.70208)
Supplement: Supplementary file 1 — Figure S1: Daily surface salinity measurements for both sampling locations from 1993 to 2021, includes all measurements from June to October. Coloured points indicate mean salinity values for the Baltic location (blue) and North Sea location (red). Data from CMEMS https://doi.org/10.48670/moi‐00021. Figure S2: (A) Schematic of experimental setup. Coloured circles indicate experimental cups each containing 50 adult animals with three biological replicates for each time point and treatment (roman numerals I—III). Cups were placed and sampled at random, and the order in this schematic does not reflect the actual experiment. Experimental animals in each cup were divided into two pseudo‐replicates with only one of them being forwarded for RNAseq and one as back‐up in case of unsuccessful extractions. (B) Picture of the acclimation tanks, Baltic samples are marked with green tape. (C) Experimental tank after sampling t1; 7 PSU treatment is marked in blue, 15 PSU treatment is marked in black. Figure S3: Respiration rates of Acartia tonsa individuals from the Baltic Sea (blue) and the North Sea (red), standardised per dry body weight. Measurements were conducted after 16 h of acclimation to the treatment salinities. Figure S4: Naupliar survival of Acartia tonsa from the Baltic (blue) and North Sea (red) at two treatment salinities (7 and 15 PSU); points indicate mean survival at sampling days, error bars indicate standard deviations. Figure S5: Egg production (top panel) and hatching success (bottom panel) of Acartia tonsa from the Baltic (blue) and North Sea (red) after acute transfer to three treatment salinities (5, 10, 15 PSU). Figure S6: PCA of all 25,567 genes that remained passed filtering; the Baltic population is coloured in blue, the North Sea population in red. Left: ellipses show clustering by population and time; right: ellipses show clustering by population and treatment. Figure S7: Differentially expressed genes that were shared between North and Baltic Sea ( [file MEC-35-e70208-s002.docx]

**Supplements: Local Adaptation and Osmoregulatory Mechanisms of the Copepod *Acartia tonsa* Under Low Salinity Stress**

Alexandra Hahn^1*^, Jennifer C. Nascimento-Schulze^1^, Georgia Avgerinou^1^, Till Bayer^1^, Reid S. Brennan^1*^

^1^ GEOMAR Helmholtz Centre for Ocean Research Kiel
* Corresponding authors AH: ahahn@geomar.de, RSB: reid.brennan@gmail.com

**Supplementary Materials**

To verify species and clade identity, we sequenced the mtCOI region of random cultured animals originating from both sampling locations. DNA was extracted from four North Sea individuals and five Baltic Sea individuals, all preserved in ethanol. We followed the extraction protocol described in Hahn and Brennan (2024). Phylogenetic mapping against known sequences following Figueroa et al. (2020) as described in Hahn & Brennan (2024), revealed that all samples belonged to *Acartia tonsa* clade X (see Fig. S10).

**Supplementary Figures**


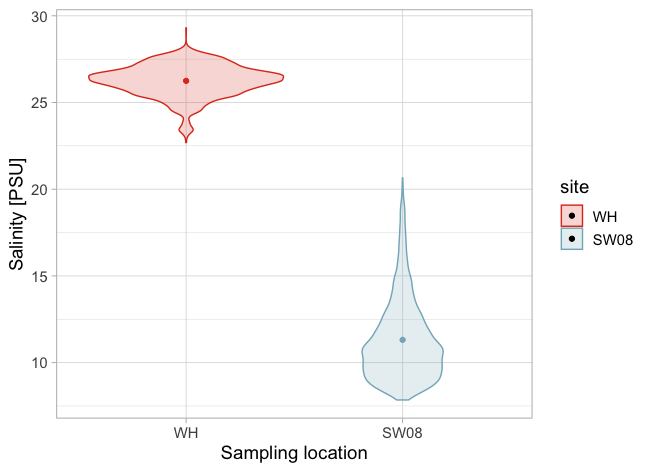


Figure S1. Daily surface salinity measurements for both sampling locations from 1993-2021, includes all measurements from June to October. Colored points indicate mean salinity values for the Baltic location (blue) and North Sea location (red). Data from CMEMS <https://doi.org/10.48670/moi-00021>


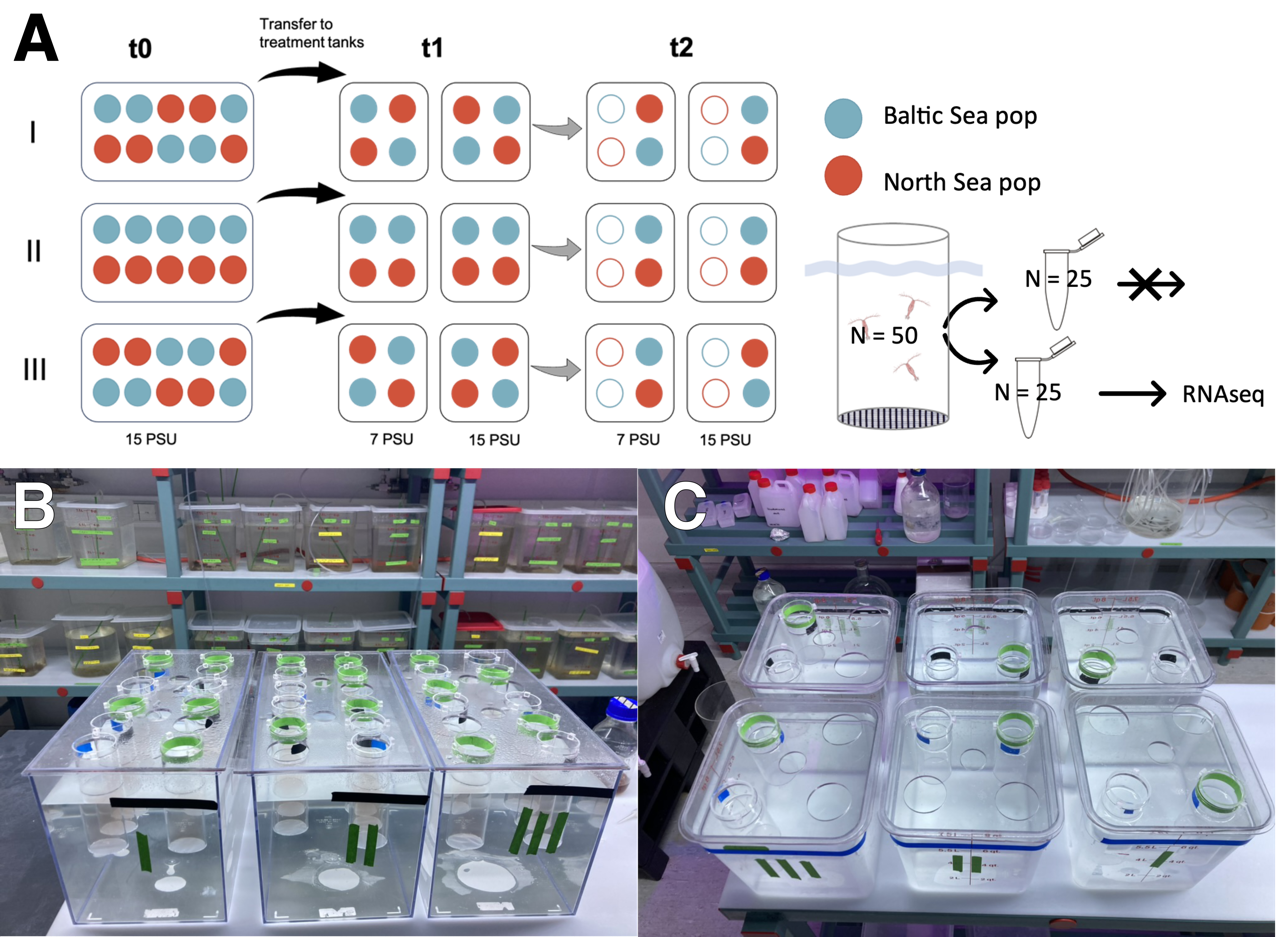


Figure S2. A) Schematic of experimental setup. Colored circles indicate experimental cups each containing 50 adult animals with three biological replicates for each time point and treatment (roman numerals I – III). Cups were placed and sampled at random and the order in this schematic does not reflect the actual experiment. Experimental animals in each cup were divided into two pseudo replicates with only one of them being forwarded for RNAseq and one as back-up in case of unsuccessful extractions. B) Picture of the acclimation tanks, Baltic samples are marked with green tape. C) Experimental tank after sampling t1, 7 PSU treatment is marked in blue, 15 PSU treatment is marked in black.


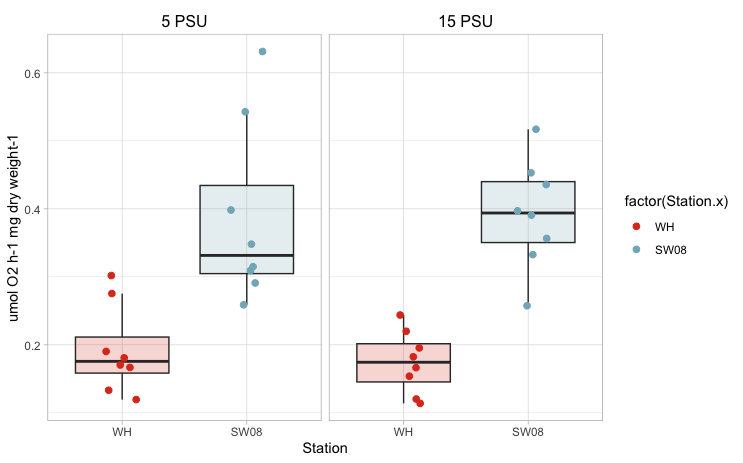


Figure S3. Respiration rates of *Acartia tonsa* individuals from the Baltic Sea (blue) and the North Sea (red), standardized per dry body weight. Measurements were conducted after 16 hours of acclimation to the treatment salinities.


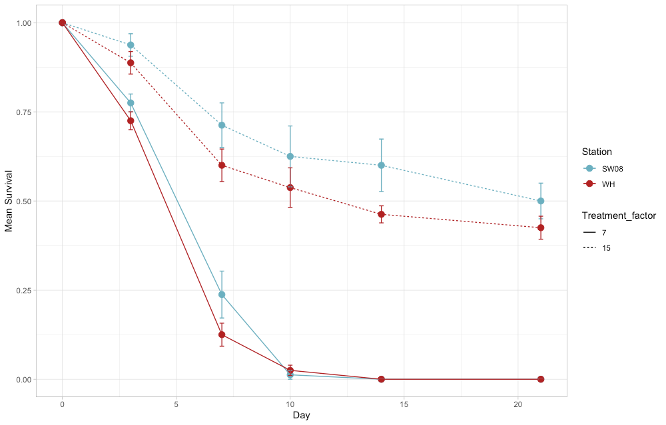


Figure S4. Naupliar survival of Acartia tonsa from the Baltic (blue) and North Sea (red) at two treatment salinities (7 and 15 PSU), points indicate mean survival at sampling days, error bars indicate standard deviations.


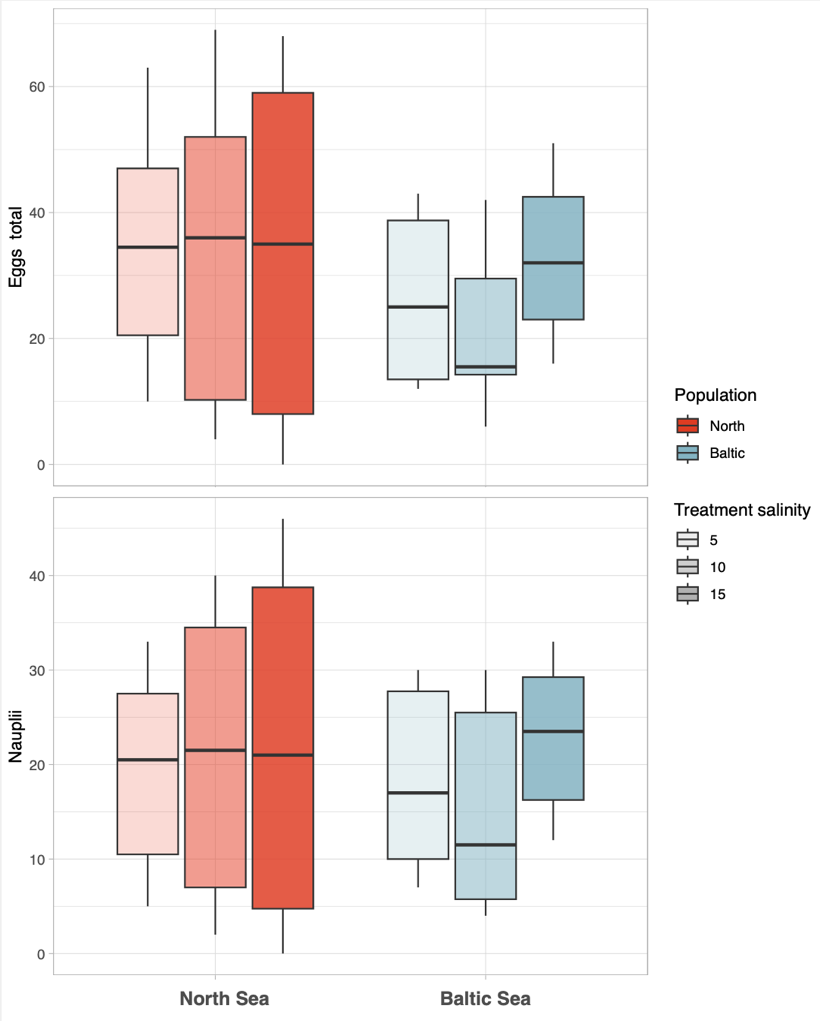


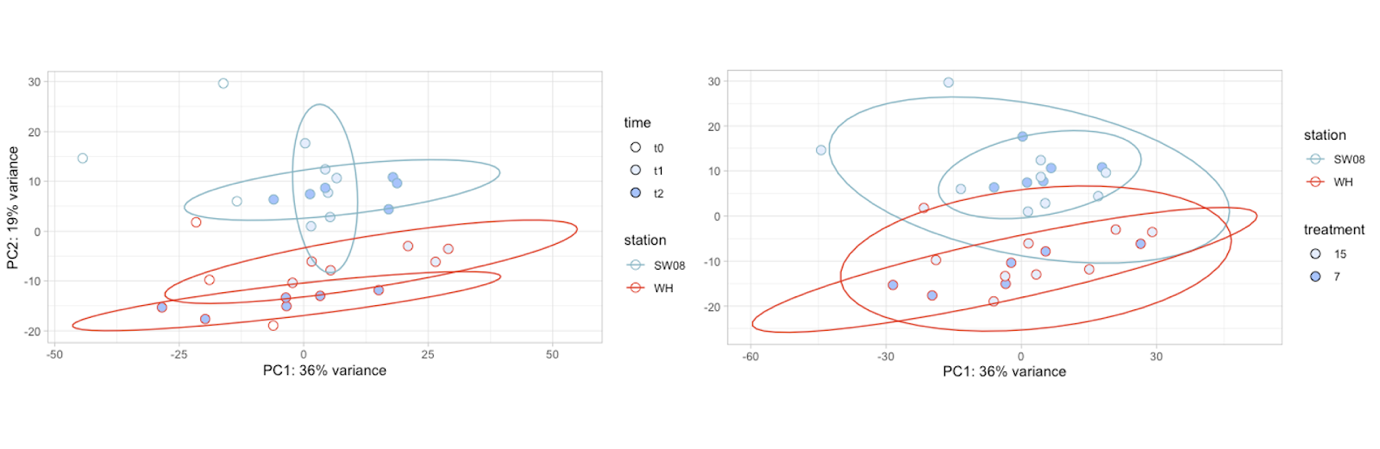
Figure S5. Egg production (top panel) and hatching success (bottom panel) of Acartia tonsa from the Baltic (blue) and North Sea (red) after acute transfer to three treatment salinities (5, 10, 15 PSU).

Figure S6. PCA of all 25,567 genes that remained passed filtering, the Baltic population is colored in blue, the North Sea population in red. Left: ellipses show clustering by population and time; Right: ellipses show clustering by population and treatment.

Figure S7. Differentially expressed genes that were shared between North and Baltic Sea (154 DEGs) clustered by expression patterns (minimum gene count per cluster = 10), clustering done using *degPatterns* from *DEGreport* (Pantano, 2022), Baltic Sea shown in blue, North Sea in red.


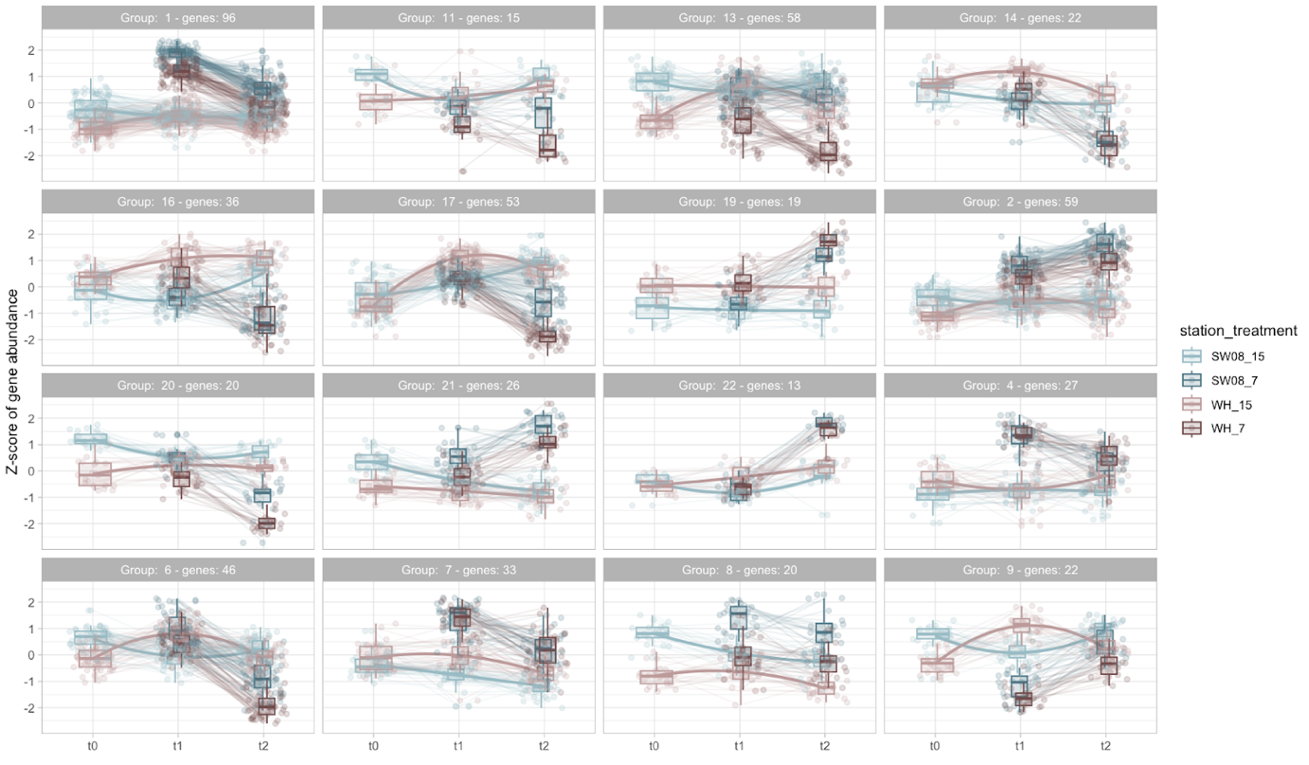


Figure S8. Salinity genes that were differentially expressed in North Sea and/or Baltic Sea samples (602 DEGs, including the 154 shared DEGs shown in Figure S7) show patterns in relative expression (minimum gene count per cluster = 10), clustering done using *degPatterns* from *DEGreport* (Pantano, 2022), Baltic Sea shown in blue, North Sea in red.


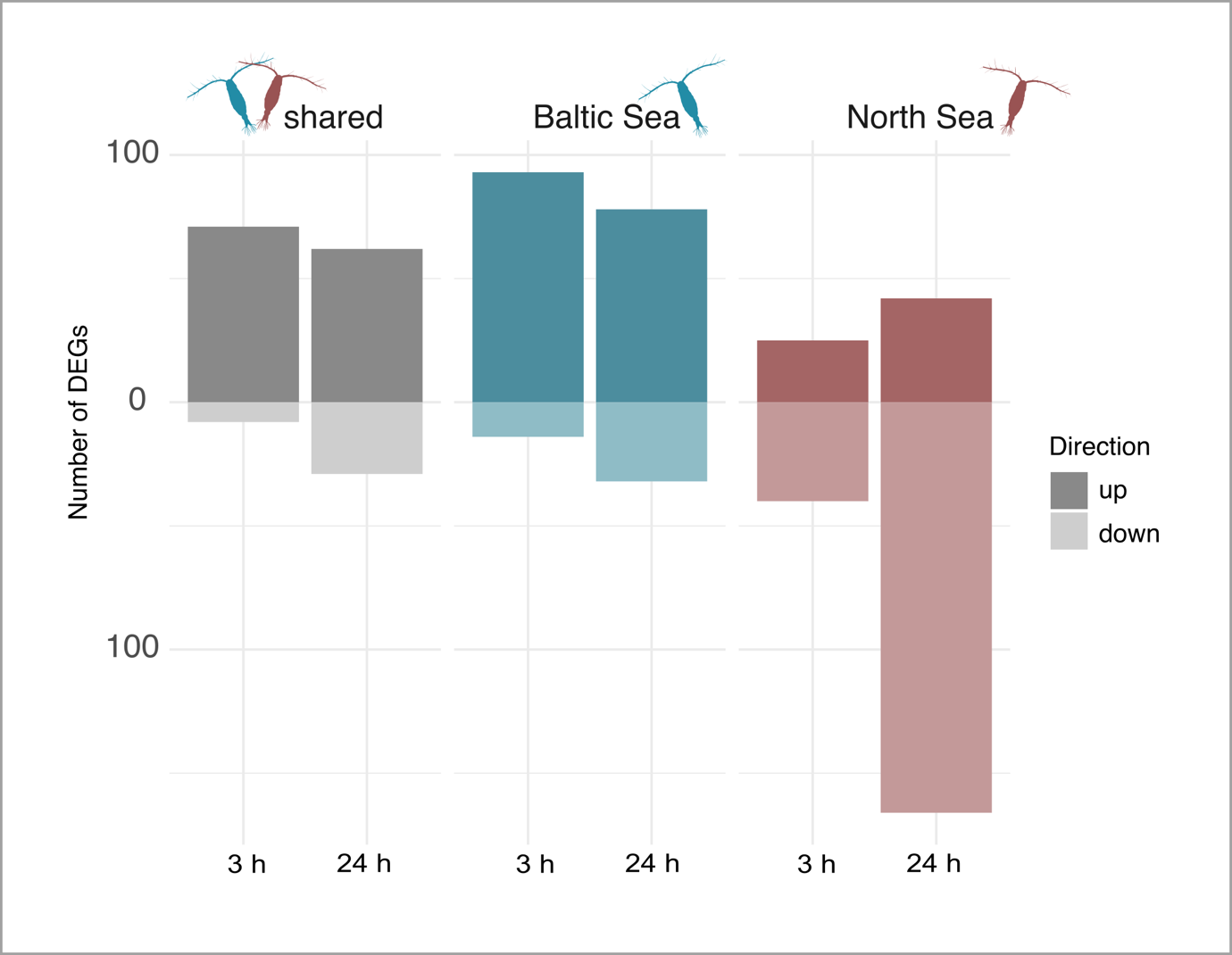


Figure S9. Differentially expressed salinity genes identified by pairwise comparisons in *DESeq2* (Love et al., 2014). Over-expressed genes have positive values assigned (log_2_ fold change > 0, dark colors), and under-expressed genes have negative values assigned (log_2_ fold changes < 0, light colors). Population-specific DEGs for the Baltic Sea are shown in blue, for the North Sea in red and DEGs shared between both populations in grey.

Figure S10. Phylogenetic tree of genotyped North and Baltic Sea individuals mapped to sequences of known species and clade identity. Mapping of mtCOI sequences followed a Bayesian approach. Baltic samples are shown in blue, North Sea samples in red.

Figueroa, N. J., Figueroa, D. F., & Hicks, D. (2020). Phylogeography of *Acartia tonsa* Dana, 1849 (*Calanoida: Copepoda*) and phylogenetic reconstruction of the genus *Acartia* Dana, 1846. *Marine Biodiversity*, *50*(2). <https://doi.org/10.1007/s12526-020-01043-1>

Hahn, A., & Brennan, R. S. (2024). Phenotypic plasticity drives seasonal thermal tolerance in a Baltic copepod. *Journal of Experimental Marine Biology and Ecology*, *576*. <https://doi.org/10.1016/j.jembe.2024.152014>

Love, M. I., Huber, W., & Anders, S. (2014). Moderated estimation of fold change and dispersion for RNA-seq data with DESeq2. *Genome Biol*, *15*(12), 550. <https://doi.org/10.1186/s13059-014-0550-8>

Pantano, L. (2022). *DEGreport: Report of DEG analysis*. In (Version R package version 1.34.0) <http://lpantano.github.io/DEGreport/>
